# Antioxidant activities of *Clerodendrum cyrtophyllum* Turcz leaf extracts and their major components

**DOI:** 10.1371/journal.pone.0234435

**Published:** 2020-06-23

**Authors:** Jing Zhou, Qi Yang, Xiaochen Zhu, Tong Lin, Dongdong Hao, Jing Xu

**Affiliations:** 1 Key Laboratory of Advanced Materials of Tropical Island Resources of Ministry of Education, School of Chemical Engineering and Technology, Hainan University, Haikou, P. R. China; 2 School of Life and Pharmaceutical Sciences, Hainan University, Haikou, P. R. China; 3 School of Ecology and Environment, Hainan University, Haikou, P. R. China; Institute for Biological Research "S. Stanković", University of Belgrade, SERBIA

## Abstract

This study was designed to investigate the antioxidant properties of the extracts and subfractions of various polarities from *Clerodendrum cyrtophyllum* Turcz leaves and the related phenolic compound profiles. The ethyl acetate fraction (EAF) showed the most potent radical-scavenging activity for DPPH radicals, ABTS radicals, and superoxide anion (O_2_^**·**-^) radicals as well as the highest reducing power of the fractions tested; the *n*-butyl alcohol fraction (BAF) was the most effective in scavenging hydroxyl radical (OH^**·**^), and the dichloromethane fraction (DMF) exhibited the highest ferrous ion chelating activity. Twelve phenolic components were identified from the EAF of *C*. *cyrtophyllum*. Additionally, acteoside (**1**) was found to be a major component (0.803 g, 0.54%) and show DPPH and ABTS radical scavenging activities with IC_50_ values of 79.65±3.4 and 23.00±1.5 μg/ml, indicating it is principally responsible for the significant total antioxidant effect of *C*. *cyrtophyllum*. Our work offers a theoretical basis for further utilization of C. *cyrtophyllum* as a potential source of natural, green antioxidants derived from plants.

## 1. Introduction

Reactive oxygen species (ROS), which comprise oxygen radicals, nonradical oxidizing species and singlet oxygen (^1^O_2_), are inevitable by-products of oxidative metabolism in all living organisms [1]. ROS are particularly unstable and can rapidly react with most biological molecules, including proteins, lipids, lipoproteins and nucleic acids; excess ROS can lead to oxidative stress and induce cellular damage or tissue injury associated with ageing, atherosclerosis, carcinogenesis and mutagenesis [2]. Fortunately, the prominent antioxidant enzymes of endogenous ROS defence can efficiently protect against these harmful free radical attacks, but they are unable to prevent damage completely; thus, exogenous antioxidants are vital to maintaining health [3]. However, the usage of synthetic antioxidants has been increasingly restricted over time because of their potential health risks, such as protein or DNA damage, and toxic effects [4]. Consequently, research has focused on identifying safe, naturally occurring antioxidant alternatives to protect the human body against deterioration or to scavenge free radicals and prevent ROS-associated chronic ageing problems. Specifically, plant-induced antioxidants have been of considerable interest in recent years due to their safety and wide distribution [5].

Phenolic compounds are a large group of phytochemicals that are commonly found in both edible and inedible plants and are reported to have various biological effects, including antioxidant activity [6]. Crude extracts of herbs, spices and other plant materials rich in phenols have been putatively recognized to have medicinal properties or beneficial impacts on human health, and they are of increasing interest because they have been shown to be highly effective scavengers of a broad spectrum of oxidants and inhibitors of lipid peroxidation [7].

The Lamiaceae family includes about 7,000 species allocated in 236 genera, with almost cosmopolitan distribution, currently divided into nine subfamilies. Among these subfamilies, Ajugoideae and Viticoideae were originally parts of the Verbenaceae family, being transferred to Lamiaceae as a result of several systematic studies on the two families [8]. The species *Clerodendrum cyrtophyllum* Turcz of the family Lamiaceae (Verbenaceae) is a perennial herb that mostly grow in tropical and subtropical regions and is widely distributed in southern China, especially the coast of Hainan Island, which is the richest wild source [9].

This herb possesses a good reputation in the treatment of various human disorders, such as colds, high fever, inflammation of the throat, epidemic encephalitis, furuncles, rheumatic arthritis, carbuncles, and snakebites [10]. The potent antioxidant activities of ethanolic extracts of *C*. *cyrtophyllum* were validated in our recent investigation [11]. However, the antioxidant activities of different solvent subfractions and the phenolic components of *C*. *cyrtophyllum* have rarely been studied and are poorly understood, and the antioxidant activities of the crude extracts and fractions must be assessed prior to the isolation of the antioxidant phytochemicals from the extracts.

In the present work, the antioxidant potential of the ethanolic crude extract (ECE) and its four different solvent sub-fractions, namely, the petroleum ether fraction (PEF), ethyl acetate fraction (EAF), n-butyl alcohol fraction (BAF) and the remaining fraction (RF), of *C*. *cyrtophyllum* were measured using several methods, including radical-scavenging activities on DPPH, ABTS, superoxide anion, and hydroxyl radicals as well as ferric reducing power and ferrous ion-chelating activity. The total phenolic content (TPC) and total flavonoid content (TFC) were used to quantify the antioxidant components in the extracts. Twelve phenolic components were isolated from the EAF subfraction, and their structures were unambiguously established by comprehensive spectroscopic analyses and comparison with the literature. Furthermore, the DPPH and ABTS radical scavenging activities of the purified compounds and the correlation of these of compounds with the antioxidant potential of *C*. *cyrtophyllum* were also investigated.

## 2. Materials and methods

### 2.1. Plant material and ethics statement

Fresh *C*. *cyrtophyllum* leaves were collected from the Extinct Crater Garden (E110°13′14″, N19°55′56″) on Hainan Island, China, in March 2013. The People’s Republic of China issued the specific permissions are required from authority of plant collection in a protected area of land, but not a national geological garden. Our plant materials were collected in a national geological garden and the author was not obliged to have any permissions. This work did not involve endangered or protected species, the species *C*. *cyrtophyllum* is a common plant growing nearby the curbside. A voucher specimen of the plant (P-DQ001) was deposited in the herbarium of the Institute of Tropical Agriculture and Forestry, Hainan University, China.

### 2.2. Extraction procedure

Dried leaves of *C*. *cyrtophyllum* (150 g) were weighed and sieved (20 mesh) in an herb grinder (118 Swing, Zhejiang, China); the powdered samples were extracted twice according to a previous protocol [11]. The solvent was removed from the combined filtrates, and 61.44 g of ECE was obtained and redissolved in distilled water (500 ml). The solution was partitioned with 3×250 ml petroleum ether (60–90°C), 3×250 ml dichloromethane, 3×250 ml ethyl acetate and 3×250 ml *n*-butanol. The resulting extracts were concentrated to yield 0.65, 5.53, 4.13, 13.28 and 36.97 g of the subfractions PEF, DMF, EAF, BAF and RF, respectively. The samples were stored at 4°C. http://dx.doi.org/10.17504/protocols.io.bdawi2fe [PROTOCOL DOI]

### 2.3. Antioxidant activity

The antioxidant activities of samples were determined using standard methods. VC and BHT were used as positive standards in the radical-scavenging assays. Gallic acid was used as a positive standard in the ferric reducing power assay. Ethylene diamine tetra acetic acid (EDTA) was used as a positive standard for the ferrous ion-chelating activity assay.

#### 2.3.1. DPPH radical-scavenging activity

The DPPH radical scavenging activities were estimated [11] by mixing 0.1 ml of the extract with 3.9 ml of 60 μM solution of DPPH in ethanol. After 30 min of reaction, the absorbance was measured at 517 nm. The inhibition percent and 50% inhibition (IC_50_) values of DPPH radicals were calculated. http://dx.doi.org/10.17504/protocols.io.jiqckdw [PROTOCOL DOI]

#### 2.3.2. ABTS radical-scavenging activity

The method described by [11] was used to determine the ABTS radical-scavenging capacity. An aliquot of extract (0.1 ml) was added to 3.9 ml of ABTS radical solution. The mixture was reacted for 30 min, and the absorbance at 734 nm was measured. The inhibition percent and IC_50_ values of the extracts for ABTS radical were calculated. http://dx.doi.org/10.17504/protocols.io.jirckd6 [PROTOCOL DOI]

#### 2.3.3. Superoxide radical-scavenging activity

The superoxide radical scavenging effects were examined [12]. Briefly, 1 ml of the extract was added to 1 ml of 50 μM NBT solution, 1 ml of 468 μM NADH, and a 1 ml aliquot of 60 μM PMS reaction mixture. After 5 min, the absorbance was read at 560 nm. The inhibition percent and IC_50_ values were calculated. http://dx.doi.org/10.17504/protocols.io.bdaxi2fn [PROTOCOL DOI]

#### 2.3.4. Hydroxyl radical-scavenging activity

The scavenging of hydroxyl radicals was determined following the method of Guo et al. [12]. The reactions were performed with 0.3 ml of 20 mM sodium salicylate, 2.0 ml of 1.5 nM FeSO_4_, 1.0 ml of sample, and 1.0 ml of 6 mM H_2_O_2_. The reaction mixture was incubated for 1 h at 37°C. The absorbance was measured at 510 nm. The inhibition percent and 50% of absorbance (EC_50_) were calculated. http://dx.doi.org/10.17504/protocols.io.bdazi2f6 [PROTOCOL DOI]

#### 2.3.5. Reducing power

The reducing power of the samples were assayed using the method of Guo et al. [12] Briefly, 1 ml of extract was added to 2.5 ml of phosphate buffer (0.2 M, pH 6.6) and 2.5 ml of 1% potassium ferricyanide. After 20 min, 2.5 ml of 10% trichloroacetic acid (TCA) was added, and then the mixture was centrifuged at 3000 rpm for 10 min. The upper layer (2.5 ml) was mixed with 2.5 ml of distilled water and 0.5 ml of 0.1% ferric chloride, and after 10 min, the absorbance was measured at 700 nm. The EC_50_ values were calculated from the graph of inhibition percentage against extract concentration. http://dx.doi.org/10.17504/protocols.io.bda2i2ge [PROTOCOL DOI]

#### 2.3.6. Ferrous ion-chelating activity

The ferrous ion-chelating activities were determined according to Guo et al. [12] A 1 ml aliquot of extract was added to a solution of 100 μL of FeCl_3_ (2.0 mM), 3.7 ml of distilled water and 200 μL of ferrozine (5.0 mM). After 20 min, the absorbance was recorded at 562 nm. The inhibition percent and IC_50_ values were calculated. http://dx.doi.org/10.17504/protocols.io.bda4i2gw [PROTOCOL DOI]

#### 2.3.7. Total phenolic content (TPC)

The TPCs in the samples were determined by a colorimetric method based on the procedure described by Zhou et al. [11] Folin-Ciocalteu (FC) reagent (2 ml) was added to 2 ml of diluted extract. After 3 min, 750 μL of sodium carbonate anhydrous solution (7.5%, w/v) was added, and the mixture was adjusted to 10 ml with distilled water. After 2 h, the absorbance was recorded at 765 nm. Calibration curves were constructed with gallic acid as the standard at concentrations ranging from 0–100 μg/ml. http://dx.doi.org/10.17504/protocols.io.bda5i2g6 [PROTOCOL DOI]

#### 2.3.8. Total flavonoid content (TFC)

The amounts of total flavonoids were quantified [11]. The reaction mixture consisted of 1.0 ml of extract, 0.3 ml of 5% sodium nitrite and 4 ml of 60% ethanol. After 6 min, 0.3 ml of 10% aluminium nitrite was added. After 6 mins, 4 ml of 1 M sodium hydroxide solution was added. Then, the volume was brought to 10 ml, and the absorbance was measured at 510 nm. The TFC was calculated and is expressed as rutin equivalents (RE). A calibration curve was constructed with different concentrations of rutin (15–75 μg/ml) as a standard. dx.doi.org/10.17504/protocols.io.bdbdi2i6 [PROTOCOL DOI]

### 2.4. Isolation of the antioxidant metabolites from the EAF

**Fig 1:**
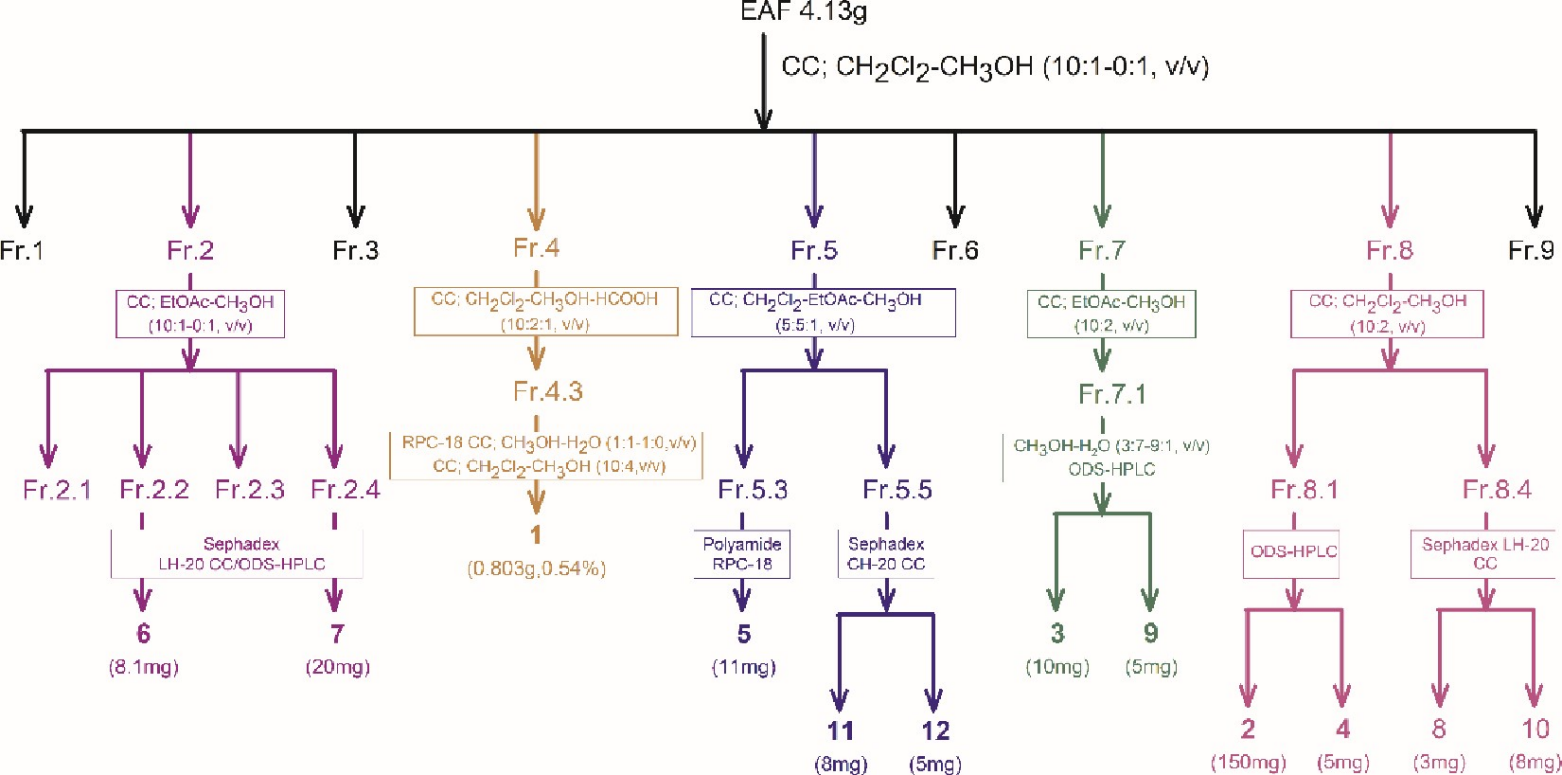
The fractionation of the ethyl acetate fraction (EAF) by sequential separation with solvents and column chromatography.

EAF (4.13 g), which showed the strongest antioxidant activity, was subjected to silica gel column chromatography (CC), employing a step gradient of CH_2_Cl_2_-CH_3_OH (10:1, 10:2, 10:3, 10:5, 1:1, 0:1, v/v), and afforded eleven fractions (Fr. 1-Fr. 9) (Fig 1). Fr. 2 was subjected to open silica gel CC using gradient elution with EtOAc-CH_3_OH (10:1–0:1, v/v) to yield fractions Fr. 2.1–2.4. Fr. 2.2 and 2.4 were separated using Sephadex LH-20 CC/ODS-HPLC to afford **6** (8.1 mg, 0.54 ‱) and **7** (20 mg, 1.33 ‱), respectively. Fr. 4 was subjected to polyamide CC using CH_2_Cl_2_-CH_3_OH-HCOOH as the eluent (10:2:1, v/v). Promising subfraction Fr. 4–3 was separated by RP C-18 CC eluted with CH_3_OH-H_2_O (1:1–1:0, v/v). Final purification was achieved by polyamide CC using CH_2_Cl_2_-CH_3_OH (10:4, v/v) to yield **1** (0.803 g, 0.54%). Fr. 5 was subjected to polyamide CC with CH_2_Cl_2_-EtOAc-CH_3_OH (5:5:1, v/v) as the eluent. Fr. 5–3 and Fr. 5–5 were separated using polyamide/RP C-18/Sephadex LH-20 CC to yield **5** (11 mg, 0.73 ‱), **11** (8 mg, 0.53 ‱) and **12** (5 mg, 0.33 ‱). Fr. 7.1, collected from Fr. 7 was subjected to polyamide CC with EtOAc-CH_3_OH (10:2, v/v), followed by ODS-HPLC using a gradient of CH_3_OH-H_2_O (3:7–9:1, v/v) as the eluent to yield **3** (10 mg, 0.66 ‱) and **9** (5 mg, 0.33 ‱). Fr. 8–1, Fr. 8–3 and Fr. 8–4, obtained from Fr. 8 with CH_2_Cl_2_-CH_3_OH (10:2, v/v), were separated using ODS-HPLC/Sephadex LH-20 CC to yield **2** (150 mg, 0.1%), **4** (5 mg, 0.33 ‱), **8** (3 mg, 0. 20 ‱) and **10** (8 mg, 0.53 ‱).http://dx.doi.org/10.17504/protocols.io.bda7i2hn [PROTOCOL DOI]

### 2.5. Statistical analysis

Triplicate analyses were performed, and the results are presented as the mean ± standard deviation. Each experiment was performed three times. Statistical analyses were performed using ANOVA; *p* < 0.05 indicated significance. This analysis was carried out using Sigmaplot (Version 13.0).

## 3. Results and discussion

### 3.1. Scavenging effects on DPPH free radical

**Fig 2:**
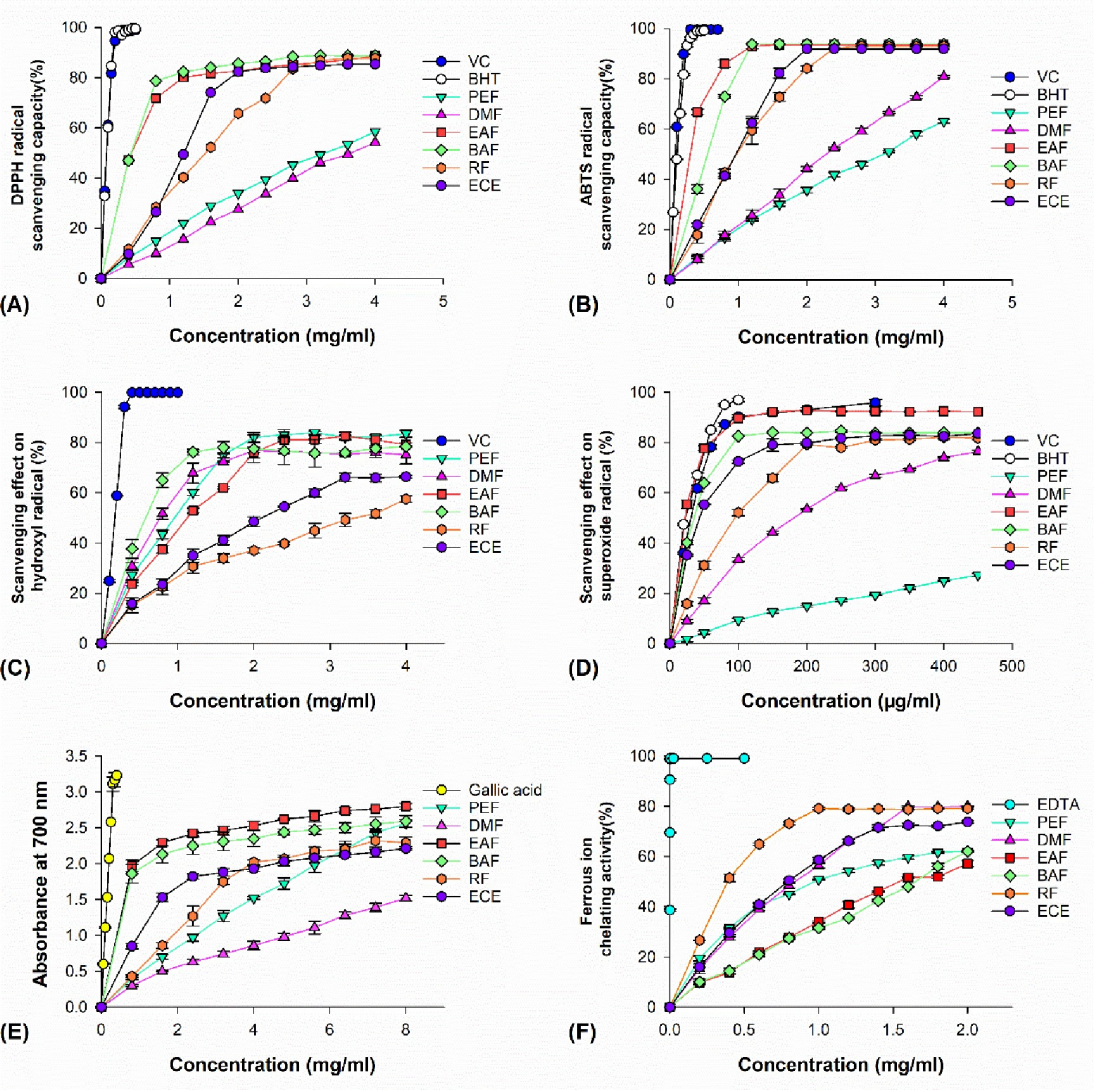
**The antioxidant and free-radical scavenging activities of the extracts and fractions of *C*. *cyrtophyllum* leaves evaluated by using six *in vitro* antioxidant models, namely, the (A) DPPH·, (B) ABTS·, (C)** OH·, **and (D) O_2_^·-^ radical scavenging activities as well as the (E) reducing power and (F) iron-chelating capacity.** ECE, **ethanolic crude extract;** PEF, **petroleum ether fraction; EAF, ethyl acetate fraction; BAF, *n*-butyl alcohol fraction; RF, remaining fraction. Responses are the means ± SD (n = 3)**.

DPPH radical quenching assays are commonly used for the determination of antioxidant activities, and an antioxidant candidate that proves promising in the scavenging of DPPH radical may inhibit one of the many mechanisms by which oxidative stress is caused by lipid peroxidation [13]. To evaluate the radical-scavenging abilities of the various extracts of *C*. *cyrtophyllum* in our specific experimental setup, we found a significant (*p<*0.05) dose-dependent decrease in the concentration of DPPH due to the scavenging activities of the extracts (Fig 2A). As illustrated in Table 1, EAF and BAF showed the lowest IC_50_ values (0.36 mg/ml), corresponding to the greatest DPPH radical-scavenging capacities, followed by RF (IC_50_ was 1.33 mg/ml). DMF was found to exert the weakest radical-scavenging effect and show the highest IC_50_ value (4.04 mg/ml). The DPPH radical-scavenging activity tended to decrease in the following order: VC>BHT>EAF>BAF>ECE>PEF>RF>DMF. However, none of the *C*. *cyrtophyllum* extracts were more effective than the positive standards, VC and BHT (IC_50_ values of 0.07 and 0.08 mg/ml, respectively).

**Table 1:** Antioxidant activities and contents of total phenolics and total flavonoids of the ethanolic extracts and subfractions from *C*. *cyrtophyllum*.

	DPPH IC_50_ (μg/ml)	ABTS IC_50_ (μg/ml)	OH IC_50_ (μg/ml)	FRAP EC_50_ (μg/ml)	Chelating IC_50_ (μg/ml)	Superoxide IC_50_ (μg/ml)	TFC (mg RE/g DW)	TPC (mg GAE/g DW)
ECE	1.20±0.02	0.89±0.01	1.99±0.01	0.47±0.02	0.91±0.00	44.83±0.63	93.63±0.59	12.42±0.03
PEF	3.36±0.04	2.98±0.02	0.90±0.02	1.07±0.07	0.99±0.01	>400	0.41±0.00	0.07±0.01
DMF	4.04±0.08	2.03±0.02	0.84±0.04	1.56±0.04	0.74±0.01	164.25±2.45	3.47±0.04	0.66±0.00
EAF	0.36±0.01	0.10±0.05	1.07±0.01	0.20±0.01	1.63±0.05	13.06±1.56	20.44±0.17	2.08±0.00
BAF	0.36±0.01	0.55±0.02	0.65±0.08	0.22±0.02	1.65±0.02	35.08±1.51	57.52±0.13	5.10±0.02
RF	1.33±0.02	0.94±0.02	3.41±0.32	0.93±0.04	0.43±0.01	94.49±2.20	6.33±0.02	3.90±0.02
BHT	0.08±0.00	0.10±0.00	ND	ND	ND	26.73±0.59	ND	ND
VC	0.07±0.00	0.06±0.00	0.17±0.01	ND	ND	22.22±0.31	ND	ND
Gallic acid	ND	ND	ND	0.05±0.41	ND	ND	ND	ND
EDTA	ND	ND	ND	ND	0.16±0.01 (μg/ml)	ND	ND	ND

Consistent with previous reports, DPPH radicals could be effectively inhibited by the ethanol extract of *C*. *infortunatum* leaves with an inhibition of 92.6% at the tested concentration (250 μg/ml), whereas the chloroform and petroleum ether extracts exhibited low DPPH scavenging activities, and their percentage inhibitions were 52.2% and 16.7% at the same concentration [14]. The scavenging ability of *C*. *cyrtophyllum* is not very remarkable when compared to those of other medicinal *Clerodendrum* plants.

### 3.2. Scavenging effects on ABTS free radical

The relatively stable ABTS radical is recommended for use in the determination of antioxidant activity of plant extracts, as the colour of plant extracts do not interfere in this determination [15]. The efficiency of various *C*. *cyrtophyllum* extracts to scavenge ABTS radicals was increased significantly (*p<*0.05) with increasing concentration of the extract (Fig 2B). At 0.8 mg/ml, the scavenging activities of the extracts for ABTS radicals decreased in the following order: EAF (86.1%) > BAF (72.9%) > RF (42.6%) > ECE (41.3%) > DMF (17.7%) > PEF (16.7%). At 1.6 mg/ml, the rank of the antioxidant activities was as follows: EAF (93.5%) > BAF (93.9%) > ECE (82.3%) > RF (72.8%) > DMF (33.7%) > PEF (29.9%). RF achieved 93.1% inhibition of ABTS radicals, which was more than PEF (63.1%), at the highest tested concentration (4.0 mg/ml). As indicated in Table 1, the IC_50_ values of all the examined fractions indicated that the ABTS radical-scavenging ability of EAF was greatest, as it showed the lowest IC_50_, 0.10 mg/ml, which was almost the same as that of the positive standards, VC and BHT (IC_50_ values of 0.06 and 0.10 mg/ml, respectively).

With respect to other extracts, the aqueous and hydroalcoholic extracts of *Verbena officinalis* demonstrated ABTS radical-scavenging capacities with IC_50_ values of 99.27 and 301.11 μg/ml, respectively [16], indicating that *C*. *cyrtophyllum* is less effective than other *Verbenaceae* plants, but EAF of *C*. *cyrtophyllum* is more effective in comparison.

### 3.3. Superoxide radical-scavenging activity

Although the superoxide radical is not a highly reactive oxidative species that is toxic to cellular components, its dismutation can result in dangerous hydroxyl radical formation via Fenton-type chemistry and induce lipid peroxidation [17]. The ECE and five subfractions of *C*. *cyrtophyllum* exhibited linear concentration-dependent inhibition of superoxide radicals (Fig 2C). As shown in Table 1, EAF exhibit the strongest superoxide radical-scavenging ability with the lowest IC_50_ value, 13.06 μg/ml, which is better than those of positive standards VC and BHT (IC_50_ values of 22.22 and 26.73 μg/ml, respectively). The IC_50_ values were in the following order: EAF > VC > BHT > BAF > ECE > RF > DMF > PEF. The PEF subfractions of various plants generally show the weakest free radical scavenging activities, which could be because few lipophilic extracts are capable of showing antioxidant activity. EAF was the most effective superoxide radical scavenger, indicating that the potential antioxidant compounds in *C*. *cyrtophyllum* were of medium polarity.

With respect to other extracts, the ethanolic extracts of *Stachytarpheta angustifolia* showed superoxide radical inhibitions in a range of 73.3%-80.8% at concentrations of 125–250 μg/ml, and the IC_50_ value was 64.68 μg/ml [18]. *C*. *cyrtophyllum* extracts, especially the EAF subfraction, might exhibit better superoxide radical scavenging ability than *S*. *angustifolia*.

### 3.4. Hydroxyl radical-scavenging activity

Among oxygen-centred radicals, hydroxyl radicals are the most chemically reactive, and they therefore easily react with biomolecules such as proteins, lipids, and nucleic acids in almost every biological membrane, causing cell damage and hence resulting in ageing, cancer and several other diseases [19]. In this study, the Fe^2+^/H_2_O_2_ system was used to generate hydroxyl radicals and measure the scavenging activities of ECE and five subfractions. The scavenging activities of the various extracts on hydroxyl radicals increased quickly with increasing concentration (Fig 2D). In Table 1, we present the IC_50_ values of the various *C*. *cyrtophyllum* extracts and the positive control (VC) for the scavenging of hydroxyl radicals, and the values were 1.99 mg/ml, 0.90 mg/ml, 0.84 mg/ml, 1.07 mg/ml, 0.65 mg/ml, 3.41 mg/ml and 0.17 mg/ml for ECE, PEF, DMF, EAF, BAF, RF, and VC, respectively. The scavenging activities for hydroxyl radicals in descending order were VC > BAF > DMF > PEF >EAF > ECE >RF.

The ethanolic extracts of *Vitex negundo* exhibited dose-dependent scavenging and quenched approximately 40.2% and 56.7% of hydroxyl radicals at concentrations of 0.5 mg/ml and 1.2 mg/ml, respectively [20]. Apparently, *C*. *cyrtophyllu* extracts could be used to obtain effective hydroxyl radical scavengers that can help prevent oxidative damage; their effect is weaker than that of *S*. *angustifolia* but comparable to that of *V*. *negundo*.

### 3.5. Reducing power

Several investigations have indicated that the reducing powers of certain plant extracts are related to their antioxidant activity. The presence of reducers (i.e., antioxidants) can reduce the Fe^3+^/ferricyanide complex to the ferrous form by donating an electron, concomitantly decreasing the oxidized intermediates from lipid peroxidation processes [21]. The ECE and its five subfractions showed dose-dependent reducing powers (0–8 mg/ml) (Fig 2E). As shown in Table 1, the EC_50_ values of reducing power were found to be 0.203 mg/ml and 0.216 mg/ml for EAF and BAF, respectively, while the other subfractions all exhibited lower reducing powers varying from 0.473 to 1.562 mg/ml. The EC_50_ value of gallic acid was 0.045 mg/ml. The obtained results suggest that gallic acid has better reducing ability than antioxidants from *C*. *cyrtophyllum* extracts.

The ethanolic extracts of *C*. *inerme* and *Lantana camara* were examined previously and compared to *C*. *cyrtophyllum*. The extracts of *C*. *inerme* presented reducing powers of 0.79 (2.5 mg/ml) and 0.86 (5.0 mg/ml) [22]. The ECE and subfractions BAF and EAF obtained from *C*. *cyrtophyllum* had better antioxidant properties than those of the other *Verbenaceae* plants described previously, meaning that it might have a higher content of reductants bearing aromatic hydroxyl groups that can interrupt free radical chain reactions by hydrogen donation.

### 3.6. Ferrous ion-chelating activity

Ferrous ions are regarded as precursors of reactive oxidants, and these ions can catalyse lipid peroxidation *via* Fenton and Haber-Weiss reactions, resulting in the generation of hydroxyl radicals. Chelating ability is regarded as a significant indicator of potential antioxidant activity, and this parameter can be quantified based on the absorbance of the red colour generated by the reduction of Fe^2+^ in the ferrozine complex, as the co-existing chelator may capture the ferrous ion before complex formation [23]. In this assay, both the extracts and EDTA decreased the absorbance in a dose-dependent manner (Fig 2F). The IC_50_ values of the ECE and the various subfractions for ferrous binding ranged from 0.43 to 1.65 mg/ml, which were lower than that of the positive standard (EDTA, IC_50_ of 0.16 μg/ml), suggesting that EDTA had the strongest chelating ability, as shown in Table 1.

With regard to methanolic extracts, the percentages of inhibition achieved by the leaf, stem and root extracts of *C*. *viscosum* were 9.6%, 15.8% and 5.0% at a concentration of 120 μg/ml, corresponding to IC_50_ values were between 0.68 mg/ml and 1.10 mg/ml [24]. It seems that the ferrous chelating ability of the *C*. *cyrtophyllu* extracts were greater than that of *C*. *viscosum*, and these extracts were better able to stabilize the metal ion and oxidize it.

### 3.7. Antioxidant components

#### 3.7.1 Total flavonoids and total phenolic content

The antioxidant activity of plant extracts is mainly linked to the active phytochemicals ubiquitous in plants [21]. The efficiency of phenol-based antioxidants is based on their redox properties, which allow them to quench oxygen-derived free radicals and are associated with their structural characteristics, such as the number and position of hydrogen-donating hydroxyl groups and alkyl degree of the phenolic moieties. Among these compounds, flavonoids constitute a special class of phenolic compounds and are reported to scavenge or delay oxidation by oxidizing molecules by transferring a single electron to O_2_^**·**-^ and OH^**·**^ radicals [25].

**Fig 3:**
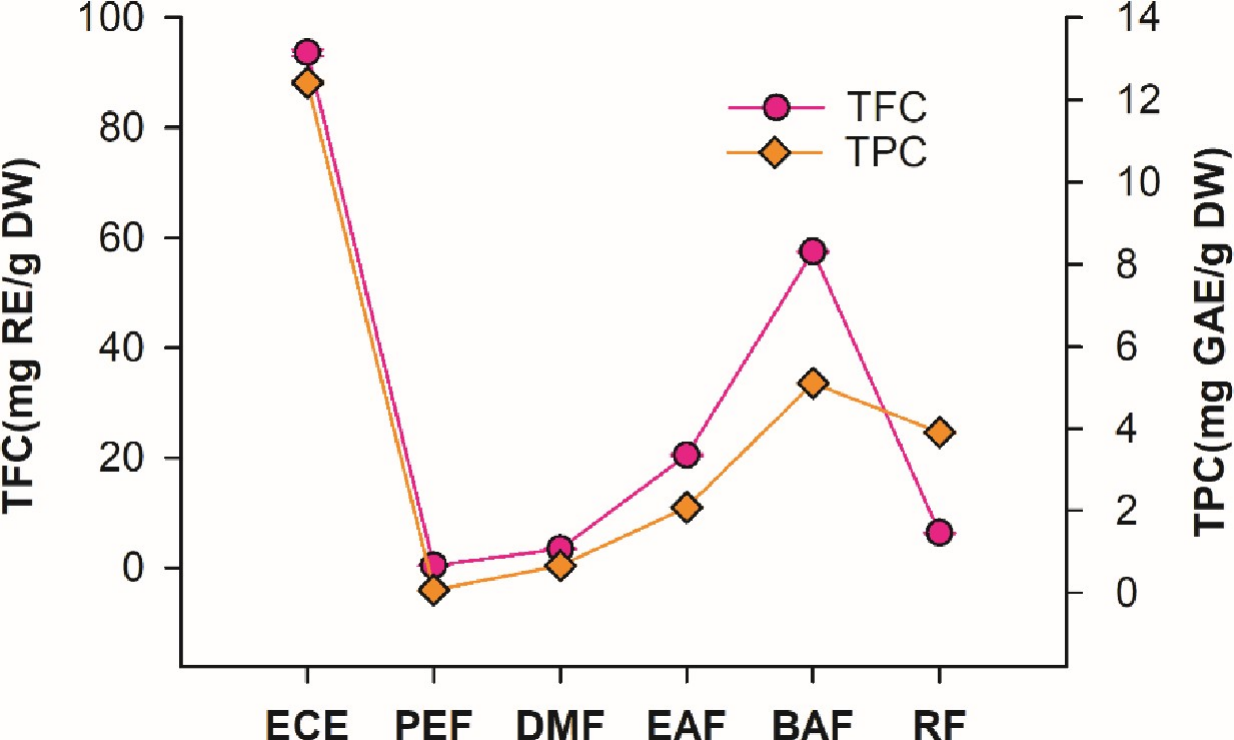
Total phenolic content (TPC) and total flavonoid content (TFC) in ECE, PEF, EAF, BAF and RF of *C*. *cyrtophyllum* leaves. Responses are the means ± SD (n = 3).

In this study, TPC and TFC were used to quantify the antioxidant compounds in the *C*. *cyrtophyllum* crude extract and fractions (Fig 3). It could be seen that the TPC results were not entirely consistent with those of TFC; BAF showed the highest phenolic and flavonoid contents, followed by EAF. These findings were consistent with a higher efficiency in radical scavenging. As Table 1 shows, the TPCs and TFCs of the test extracts varied in the ranges of 0.07–12.42 mg GAE/g DW and 0.41–93.63 mg RE/g DW. The TPCs were in the following order: ECE > BAF > RF > EAF > DMF > PEF. The TFCs were in the following order: ECE > BAF > EAF > RF > DMF > PEF. These results indicated that TPCs and TFCs of fractions obtained with solvents of various polarities were different.

The strong correlations between the total antioxidant capacities determined based on the DPPH, ABTS, OH^**·**^, O_2_^**·**-^, reducing powers and ferrous ion chelating activities with the TPCs and TFCs were observed as show in Table 1. The data indicated that the total flavonoids and phenolic contents in BAF and EAF were significantly higher than those of other fractions. EAF showed the most potent radical-scavenging activity for DPPH radicals, ABTS radicals, and superoxide anion (O_2_^**·**-^) radicals as well as the highest reducing power of the fractions tested; the *n*-butyl alcohol fraction (BAF) was the most effective in scavenging hydroxyl radical (OH^**·**^). The results are consistent with reports, which had previously suggested that the antioxidant activities of medicinal plants were mainly contributed by the phenolic compounds and flavonoids in the extracts and therefore could play an important role in the beneficial effects of corresponding important medicinal plants [26, 27]. However, DMF exhibited the highest ferrous ion chelating activity, suggesting that other components with non-phenolic hydroxyl groups in the extracts were more effective chelators of ferrous ions than phenolic compounds [28, 29]. Therefore, the phenolic and flavonoid constituents present in spices corresponding the antioxidant activities require further investigation.

#### 3.7.2 Isolation and identification of compounds 1–12

**Fig 4:**
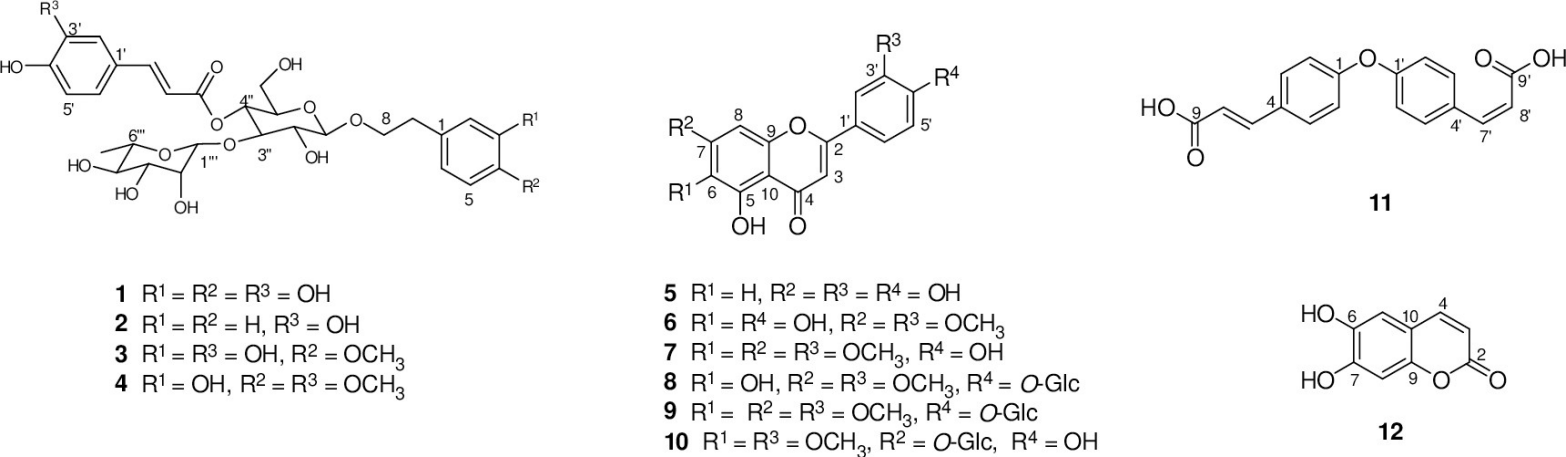
The structures of compounds 1–12 isolated from the ethyl acetate fraction (EAF) of *C*. *cyrtophyllum* leaves.

The EAF subfraction was subjected to successive separations and purifications using silica gel, polyacrylamide gel and Sephadex LH-20 gel column chromatography and semipreparative HPLC to yield compounds **1–12**. The structures (Fig 4) of all these compounds were unequivocally determined by extensive NMR spectroscopic experiments as well as mass spectrometry and comparison with data reported in the literature [30–32].

Acteoside (**1**): yellow, amorphous powder (MeOH); UV (EtOH) λ_max_ 227, 333 nm; ^1^H NMR (400 MHz, DMSO-*d*_*6*_)*δ*_H_ Aglycone: 6.61 (1H, d, *J* = 1.9 Hz, H-2), 6.62 (1H, d, *J* = 8.0 Hz, H-5), 6.47 (1H, dd, *J* = 8.0, 1.9 Hz, H-6), 2.69 (2H, m, H-7), 3.87 (1H, dd, *J* = 16.0, 8.9 Hz, H-8a), 3.58 (1H, dd, *J* = 16.0, 8.9 Hz, H-8b); Acid moiety: 7.01 (1H, d, *J* = 1.9 Hz, H-2′), 6.74 (1H, d, *J* = 8.2 Hz, H-5′), 6.96 (1H, dd, *J* = 8.2, 1.9 Hz, H-6′), 7.44 (1H, d, *J* = 15.9 Hz, H-7′), 6.18 (1H, d, *J* = 15.9 Hz, H-8′); Glucose moiety: 4.34 (1H, d, *J* = 7.9 Hz, H-1″), 3.20 (1H, dd, *J* = 9.0, 8.0 Hz, H-2″), 3.69 (1H, t, *J* = 9.0 Hz, H-3″), 4.69 (1H, t, *J* = 9.0 Hz, H-4″), 3.46 (1H, td, *J* = 8.8, 2.2 Hz, H-5″), 3.33 (2H, m, H-6″); Rhamnose moiety: 5.01 (1H, br s, H-1‴), 3.67 (1H, m, H-2‴), 3.26 (1H, dd, *J* = 9.4, 3.0 Hz, H-3‴), 3.09 (1H, t, *J* = 9.4 Hz, H-4‴), 3.32 (1H, m, H-5‴), 0.94 (3H, d, *J* = 6.2 Hz, H-6‴); ^13^C NMR (100 MHz, DMSO-*d*_*6*_)*δ*_C_ Aglycone: 129.1 (C-1), 116.3 (C-2), 145.0 (C-3), 143.6 (C-4), 115.8 (C-5), 119.6 (C-6), 35.0 (C-7), 70.3 (C-8); Acid moiety: 125.5 (C-1′), 114.7 (C-2′), 145.6 (C-3′), 148.7 (C-4′), 115.5 (C-5′), 121.5 (C-6′), 145.6 (C-7′), 113.6 (C-8′), 165.7 (C-9′); Glucose moiety: 102.3 (C-1″), 74.5 (C-2″), 79.2 (C-3″), 68.8 (C-4″), 74.5 (C-5″), 60.7 (C-6″); Rhamnose moiety: 101.3 (C-1‴), 70.5 (C-2‴), 70.4 (C-3‴), 71.7 (C-4‴), 69.1 (C-5‴), 18.2 (C-6‴); ESI-MS *m/z* 647.2 [M+Na]^+^ (C_29_H_36_O_15_Na).

Jionoside C (**2**): yellow, amorphous powder (MeOH); UV (EtOH) λ_max_ 274, 341 nm; ^1^H NMR (400 MHz, DMSO-*d*_*6*_) *δ*_H_ Aglycone: 7.27~7.39 (5H, m, H-2~H-6), 2.87 (2H, td, *J* = 7.6, 3.0, H-7), 3.71 (1H, m, H-8a), 3.49 (1H, m, H-8b); Acid moiety: 7.01 (1H, br s, H-2′), 6.74 (1H, d, *J* = 8.2 Hz, H-5′), 6.97 (1H, dd, *J* = 8.2, 1.4 Hz, H-6′), 7.44 (1H, d, *J* = 15.9 Hz, H-7′), 6.19 (1H, d, *J* = 15.9 Hz, H-8′); Glucose moiety: 4.40 (2H, d, *J* = 7.8 Hz, H-1′), 3.22 (1H, m, H-2″), 3.67 (1H, m, H-3″), 4.87 (1H, d, *J* = 12.2 Hz, H-4″), 3.46 (1H, td, *J* = 8.8, 2.2 Hz, H-5″), 3.33 (1H, m, H_2_-6″); Rhamnose moiety: 5.01 (1H, br s, H-1‴), 3.67 (1H, m, H-2‴), 3.26 (1H, m, H-3‴), 3.09 (1H, t, *J* = 9.4 Hz, H-4‴), 3.32 (1H, m, H-5‴), 0.94 (3H, d, *J* = 6.2 Hz, H-6‴); ^13^C NMR (100 MHz, DMSO-*d*_*6*_) *δ*_C_ Aglycone: 137.8 (C-1), 128.2 (C-2, C-6), 127.6 (C-3, C-5), 126.1 (C-4), 35.5 (C-7), 70.5 (C-8); Acid moiety: 125.4 (C-1′), 114.7 (C-2′),145.6 (C-3′), 148.7 (C-4′), 115.7 (C-5′), 121.4 (C-6′), 125.4 (C-7′),145.6 (C-8′), 165.6 (C-9′); Glucose moiety: 101.5 (C-1″), 74.5 (C-2″), 79.0 (C-3″), 69.7 (C-4″), 74.6 (C-5″), 60.7 (C-6″); Rhamnose moiety: 101.3 (C-1‴), 70.5 (C-2‴), 70.4 (C-3‴), 68.7 (C-4‴), 18.1 (C-5‴); ESI-MS *m/z* 615.2 [M+Na]^+^ (C_29_H_36_O_13_Na).

Jionoside D (**3**): yellow, amorphous powder (MeOH); UV (EtOH) λ_max_ 288, 337 nm; ^1^H NMR (400 MHz, DMSO-*d*_*6*_) *δ*_H_ Aglycone: 6.68 (1H, br s, H-2), 6.75 (1H, d, *J* = 8.2 Hz, H-5), 6.63 (1H, d, *J* = 8.2 Hz, H-6), 2.73 (2H, m, H-7), 3.89 (1H, dd, *J* = 15.4, 8.3 Hz, H-8a), 3.63 (1H, m, H-8b), 3.72 (3H, s, 4-OCH_3_); Acid moiety: 7.02 (1H, br s, H-2′), 6.81 (1H, d, *J* = 8.3 Hz, H-5′), 6.98 (1H, br d, *J* = 8.3 Hz, H-6′), 7.45 (1H, d, *J* = 15.9 Hz, H-7′), 6.19 (1H, d, *J* = 15.9 Hz, H-8′); Glucose moiety: 4.36 (1H, d, *J* = 8.1 Hz, H-1″), 3.20 (1H, m, H-2″), 3.69 (1H, m, H-3″), 4.70 (1H, t, *J* = 9.5 Hz, H-4″), 3.42 (1H, m, H-5″), 3.33 (2H, m, H-6″); Rhamnose moiety: 5.02 (1H, br s, H-1‴), 3.67 (1H, m, H-2‴), 3.26 (1H, m, H-3‴), 3.09 (1H, t, *J* = 9.3 Hz, H-4‴), 3.32 (1H, m, H-5‴), 0.94 (3H, d, *J* = 6.2 Hz, H-6‴); ^13^C NMR (100 MHz, DMSO-*d*_*6*_) *δ*_C_ Aglycone: 131.4 (C-1), 112.4 (C-2), 146.8 (C-3), 146.1 (C-4), 116.8 (C-5), 121.9 (C-6), 36.1 (C-7), 71.7 (C-8), 56.2 (4-OCH_3_); Acid moiety: 127.3 (C-1′), 114.7 (C-2′), 149.0 (C-3′), 146.0 (C-4′), 116.4 (C-5′), 123.0 (C-6′), 146.8 (C-7′), 115.2 (C-8′), 166.0 (C-9′); Glucose moiety: 102.9 (C-1″), 75.0 (C-2″), 79.2 (C-3″), 69.2 (C-4″), 76.0 (C-5″), 61.2 (C-6″); Rhamnose moiety: 101.2 (C-1‴), 71.5 (C-2‴), 71.7 (C-3‴), 72.1 (C-4‴), 68.8 (C-5‴), 18.6 (C-6‴); ESI-MS *m/z* 637.1 [M-H]^-^(C_30_H_37_O_15_).

Martynoside (**4**): yellow, amorphous powder (MeOH); UV (EtOH) λ_max_ 280, 327 nm; ^1^H NMR (400 MHz, DMSO-*d*_*6*_) *δ*_H_ 6.61 (1H, d, *J* = 1.8 Hz, H-2), 6.79 (1H, d, *J* = 8.2 Hz, H-5), 6.63 (1H, dd, *J* = 8.2, 1.8 Hz, H-6), 2.72 (2H, m, H-7), 3.89 (1H, dd, *J* = 15.8, 7.7 Hz, H-8a), 3.62 (1H, dd, *J* = 15.8, 7.3 Hz, H-8b); Acid moiety: 7.27 (1H, s, H-2′), 6.80 (1H, d, *J* = 8.2 Hz, H-5′), 7.09 (1H, br d, *J* = 8.2 Hz, H-6′), 7.53 (1H, d, *J* = 15.9 Hz, H-7′), 6.39 (1H, d, *J* = 15.9 Hz, H-8′); Glucose moiety: 4.35 (1H, d, *J* = 7.8 Hz, H-1″), 3.21 (1H, t, *J* = 8.4 Hz, H-2″), 3.70 (1H, m, H-3″), 4.72 (1H, t, *J* = 9.5 Hz, H-4″), 3.46 (1H, m, H-5″), 3.33 (2H, m, H-6″); Rhamnose moiety: 5.03 (1H, br s, H-1‴), 3.69 (1H, m, H-2‴), 3.29 (1H, dd, *J* = 9.4, 2.8 Hz, H-3‴), 3.11 (1H, t, *J* = 9.3 Hz, H-4‴), 3.35 (1H, m, H-5‴), 0.97 (3H, d, *J* = 6.1 Hz, H-6‴); ^13^C NMR (100 MHz, DMSO-*d*_*6*_) *δ*_C_ Aglycone: 125.7 (C-1), 116.4 (C-2), 148.0 (C-3), 146.3 (C-4), 115.6 (C-5), 119.5 (C-6), 35.0 (C-7), 70.1 (C-8), 55.7 (4-OCH_3_); Acid moiety: 131.2 (C-1′), 111.2 (C-2′), 149.4 (C-3′), 146.2 (C-4′), 112.4 (C-5′), 123.1 (C-6′), 145.5 (C-7′), 114.2 (C-8′), 165.8 (C-9′), 55.8 (3″-OCH_3_); Glucose moiety: 102.4 (C-1″), 74.5 (C-2″), 79.2 (C-3″), 69.3 (C-4″), 74.6 (C-5″), 60.8 (C-6″); Rhamnose moiety: 101.2 (C-1‴), 70.6 (C-2‴), 70.5 (C-3‴), 71.8 (C-4‴), 68.7 (C-5‴), 18.6 (C-6‴); ESI-MS *m/z* 675.3 [M+Na]^+^ (C_31_H_41_O_15_).

Luteolin (**5**): pale, yellow needles (MeOH); UV (EtOH) λ_max_ 227, 347 nm; ^1^H NMR (400 MHz, DMSO-*d*_*6*_) *δ*_H_ 12.97 (1H, s, 5-OH), 10.82 (1H, s, 7-OH), 9.91 (1H, s, 4-OH'), 9.41 (1H, s, 3-OH'), 7.41 (1H, d, *J* = 2.2 Hz, H-2'), 7.38 (1H, dd, *J* = 8.2, 2.2 Hz, H-6'), 6.80 (1H, d, *J* = 8.2 Hz, H-5'), 6.66 (1H, s, H-3), 6.43 (1H, d, *J* = 2.0 Hz, H-6), 6.17 (1H, d, *J* = 2.0 Hz, H-8); ^13^C NMR (100 MHz, DMSO-*d*_*6*_) *δ*_C_ 181.7 (C-4), 164.1 (C-7), 163.9 (C-2), 161.5 (C-9), 157.3 (C-5), 149.7 (C-3'), 145.7 (C-4'), 121.5 (C-6), 119.0 (C-1'), 116.0 (C-5'), 113.4 (C-2'), 103.7 (C-3), 102.9 (C-10), 98.8 (C-6), 93.8 (C-8); ESI-MS [M-H]^-^ *m/z* 285.0 (C_15_H_9_O_6_).

Cirsilineol (**6**): yellow, amorphous powder (MeOH); UV (EtOH) λ_max_ 274, 343 nm; ^1^H NMR (400 MHz, acetone-*d*_*6*_) *δ*_H_ 12.93 (1H, s, H-5), 7.60 (1H, d, *J* = 8.2, 1.9 Hz, H-6′), 7.59 (1H, br s, H-2′), 6.96 (1H, s, H-3), 6.95 (1H, d, *J* = 7.5 Hz, H-5′), 6.94 (1H, s, H-8), 3.94 (3H, s, H-7), 3.91 (3H, s, H-3′), 3.75 (3H, s, H-6); ^13^C NMR (100 MHz, acetone-*d*_*6*_) *δ*_C_ 182.6 (C-4), 164.3 (C-2), 158.9 (C-7), 152.4 (C-5), 148.4 (C-3′), 132.1 (C-6), 152.9 (C-9), 151.2 (C-4′), 121.7 (C-1′), 120.8 (C-6′), 116.1 (C-5′), 110.5 (C-2′), 105.4 (C-10), 103.3 (C-3), 91.9 (CH-8), 60.3 (6-OCH_3_), 56.7 (7-OCH_3_), 56.3 (3′-OCH_3_); ESI-MS: *m/z* 345.1 [M+H]^+^ (C_18_H_17_O_7_).

Cirsimartin (**7**): yellow, amorphous powder (MeOH); UV (EtOH) λ_max_ 275, 332 nm; ^1^H NMR (400 MHz, DMSO-*d*_*6*_) *δ*_H_ 12.92 (1H, s, 5-OH), 10.37 (1H, s, 7-OH), 6.93 (1H, s, H-3), 7.96 (1H, d, *J* = 8.8 Hz, H-2′, H-6′), 6.94 (2H, d, *J* = 8.8 Hz, H-3′, H-5′), 6.85 (1H, s, H-8), 3.92 (3H, s, 6-OCH_3_), 3.72 (3H, s, 7-OCH_3_); ^13^C NMR (100 MHz, DMSO-*d*_*6*_) *δ*_C_ 182.4 (C-4), 164.3 (C-2), 161.4 (C-4′), 158.8 (C-7), 152.8 (C-9), 152.2 (C-5), 132.0 (C-6), 128.1 (C-2′, C-6′), 121.3 (C-1′), 116.2 (C-3′, C-5′), 105.2 (C-10), 102.8 (C-3), 91.7 (C-8), 60.2 (6-OCH_3_), 56.6 (7-OCH_3_); ESI-MS *m/z* 313.2 [M-H]^-^ (C_18_H_15_O_7_).

Cirsilineol-4′-*O*-*β*-D-glucoside (**8**): yellow, amorphous powder (MeOH); UV (EtOH) λ_max_ 274, 341 nm; ^1^H NMR (400 MHz, DMSO-*d*_*6*_) *δ*_H_ 12.87 (1H, s, 5-OH), 7.69 (1H, dd, *J* = 8.6, 2.1 Hz, H-6′), 7.63 (1H, d, *J* = 2.1 Hz, H-2′), 7.24 (1H, d, *J* = 8.6 Hz, H-5′), 7.06 (1H, s, H-3), 7.00 (1H, s, H-8), 5.36 (1H, br s, 2″-OH), 5.13 (1H, br s, 3″-OH), 5.08 (1H, br s, 4″-OH), 5.07 (1H, d, *J* = 7.6 Hz, H-1″), 4.58 (1H, t, *J* = 5.5 Hz, 6″-OH), 3.69 (1H, t, *J* = 10.8 Hz, Ha-6″), 3.93 (3H, s, 7-OCH_3_), 3.90 (3H, s, 3′-OCH_3_), 3.73 (3H, s, 6-OCH_3_), 3.46 (1H, m, H-5″), 3.43 (1H, m, Hb-6″), 3.37 (1H, m, H-3″), 3.35 (1H, m, H-2″), 3.17 (1H, m, H-4″); ^13^C NMR (100 MHz, DMSO-*d*_*6*_) *δ*_C_ 182.3 (C-4), 163.4 (C-2), 158.7 (C-7), 152.7 (C-9), 152.0 (C-5), 149.8 (C-4′), 149.2 (C-3′), 131.9 (C-6), 123.9 (C-1′), 116.6 (C-6′), 115.2 (C-5′), 110.3 (C-2′), 105.2 (C-10), 103.8 (C-3), 99.5 (C-1″), 91.7 (C-8), 77.2 (C-5″), 76.8 (C-3″), 73.1 (C-2″), 69.6 (C-4″), 60.1 (C-6″), 60.0 (6-OCH_3_), 56.5 (7-OCH_3_), 56.1 (3′-OCH_3_); ESI-MS *m/z* 529.1 [M+Na]^+^ (C_24_H_26_O_12_Na).

Cirsimarin (**9**): yellow, amorphous powder (MeOH); UV (EtOH) λ_max_ 278, 326 nm; ^1^H NMR (400 MHz, DMSO-*d*_*6*_) *δ*_H_ 12.85 (1H, s, 5-OH), 8.07 (2H, d, *J* = 8.9 Hz, H-2′, H-6′), 7.19 (2H, d, *J* = 8.9 Hz, H-3′, H-5′), 6.98 (1H, s, H-3), 6.96 (1H, s, H-8), 5.36 (1H, d, *J* = 4.7 Hz, 2″-OH), 5.11 (1H, d, *J* = 4.5 Hz, 3″-OH), 5.04 (1H, d, *J* = 5.0 Hz, 4″-OH), 5.03 (1H, d, *J* = 6.8 Hz, H-1″), 4.58 (1H, t, *J* = 5.4 Hz, 6″-OH), 3.69 (1H, m, Ha-6″), 3.92 (3H, s, 7-OCH_3_), 3.73 (3H, s, 6-OCH_3_), 3.46 (1H, m, H-5″), 3.43 (1H, m, Hb-6″), 3.38 (1H, m, H-3″), 3.34 (1H, m, H-2″), 3.17 (1H, m, H-4″); ^13^C NMR (100 MHz, DMSO-*d*_*6*_) *δ*_C_ 182.8 (C-4), 163.8 (C-2), 160.8 (C-4′), 159.2 (C-7), 153.2 (C-9), 152.5 (C-5), 132.4 (C-6), 128.7 (C-2′, C-6′), 124.3 (C-1′), 117.1 (C-3′, C-5′), 105.7 (C-10), 104.1 (C-1″), 100.3 (C-3), 92.2 (C-8), 77.7 (C-3″), 77.0 (C-5″), 73.7 (C-2″), 70.1 (C-4″), 60.5 (C-6″), 60.5 (6-OCH_3_), 57.0 (7-OCH_3_); ESI-MS: *m/z* 499.1 [M+Na]^+^ (C_23_H_24_O_11_Na).

Jaceosidin 7-*O*-*β*-D-glucoside (**10**): yellow, amorphous powder (MeOH); UV (EtOH) λ_max_ 275, 344 nm; ^1^H NMR (400 MHz, DMSO-*d*_*6*_) *δ*_H_ 12.92 (1H, s, 5-OH), 7.58 (1H, br d, *J* = 10.0 Hz, H-6′), 7.56 (1H, br s, H-2′), 7.03 (1H, s, H-8), 6.95 (1H, s, H-3), 6.92 (1H, d, *J* = 10.0 Hz, H-5′), 5.10 (1H, d, *J* = 5.7 Hz, H-1″), 3.90 (3H, s, 3′-OCH_3_), 3.79 (3H, s, 6-OCH_3_), 3.48 (2H, m, H_2_-6″), 3.47 (1H, m, H-5″), 3.35 (1H, m, H-2″), 3.34 (1H, m, H-3″), 3.20 (1H, m, H-4″); ^13^C NMR (100 MHz, DMSO-*d*_*6*_) *δ*_C_ 182.3 (C-4), 156.5 (C-7), 152.2 (C-9), 151.4 (C-4′), 148.2 (C-3′), 132.5 (C-6), 121.1 (C-6′), 121.0 (C-1′), 115.9 (C-5′), 110.3 (C-2′), 105.8 (C-10), 100.4 (C-1″), 94.5 (C-8), 77.4 (C-5″), 76.8 (C-3″), 73.2 (C-2″), 69.7 (C-4″), 60.7 (C-6″), 60.3 (6-OCH_3_), 56.0 (3′-OCH_3_). ESI-MS: *m/z* 491.0 [M-H]^-^ (C_23_H_23_O_12_).

(1-*p*-Hydorxy-*cis*-cinnamoyl)cinnamic acid (**11**): colourless, amorphous powder (MeOH); UV (EtOH) λ_max_ 228, 290, 318 nm; ^1^H NMR (400 MHz, DMSO-*d*_*6*_) *δ*_H_ 7.60 (2H, d, *J =* 8.5 Hz, H-3, H-5), 7.49 (2H, d, *J =* 8.5 Hz, H-3′, H-5′), 7.45 (1H, dd, *J =* 15.9 Hz, H-7), 6.27 (1H, d, *J =* 15.9 Hz, H-8), 6.78 (2H, d, *J =* 8.5 Hz, H-2, H-6), 6.71 (2H, d, *J =* 8.5 Hz, H-2′, H-6′), 6.62 (1H, d, *J =* 12.8 Hz, H-7′), 5.72 (1H, d, *J =* 12.8 Hz, H-8′); ^13^C NMR (100 MHz, DMSO-*d*_*6*_) *δ*_C_ 168.2 (C-9), 168.2 (C-9′), 159.6 (C-1), 158.3 (C-1′), 144.1 (C-7), 143.8 (C-7′), 132.1 (C-3, C-5), 130.0 (C-3′, C-5′), 126.1 (C-4), 125.3 (C-4′), 118.6 (C-8′), 115.8 (C-2, C-6), 115.3 (C-8), 114.8 (C-2′, C-6′); ESI-MS *m/z* 333.1 [M+Na]^+^ (C_18_H_14_O_5_Na).

Esculetin (**12**): yellow, crystalline powder (MeOH); UV (EtOH) λ_max_ 230, 298, 346 nm; ^1^H NMR (400 MHz, DMSO-*d*_*6*_) *δ*_H_ 7.84 (1H, d, *J* = 9.4 Hz, H-4), 6.96 (1H, s, H-5), 6.72 (1H, s, H-8), 6.15 (1H, d, *J* = 9.4 Hz, H-3); ^13^C NMR (125 MHz, DMSO-*d*_*6*_) *δ*_C_ 160.8 (C-2), 150.4 (C-7), 148.5 (C-9), 144.5 (C-4), 142.9 (C-6), 112.3 (C-5), 111.5 (C-3), 110.8 (C-10), 102.6 (C-8); ESI-MS *m/z* 201.1 [M+Na]^+^ (C_9_H_6_O_4_Na).

#### 3.7.3 Antioxidant tests

The antioxidant activities of isolated compounds **1–12** were evaluated by measuring their abilities to scavenge DPPH and ABTS radicals with VC as the positive control. When the radical scavenging rates were above 70% at a concentration of 200 μg/ml, the compounds were investigated to determine their IC_50_ values. As shown in Table 2, compounds **1–5** and **12** exhibited considerable radical scavenging effects by both methods. The DPPH radical scavenging abilities increased in the order martynoside (**4**) < luteolin (**5**) < jionoside D (**3**)< acteoside (**1**) < VC < jinoside C (**2**) <esculetin (**12**), whereas the ABTS radical-scavenging activities were in the following order: martynoside (**4**) < VC < jinoside C (**2**) < luteolin (**5**) ~ jionoside D (**3**) ~acteoside (**1**) < esculetin (**12**). The *ortho*-dihydroxylated isocoumarin component, esculetin (**12**), exhibited the highest scavenging activity for DPPH and ABTS, with IC_50_ values of 47.91±0.77 and 5.88±0.51 μg/ml, respectively, indicating that it is potent than the positive control (VC, IC_50_ values of 73.14 and 57.53 μg/ml). In accordance with our previous studies, positive correlations were observed between the DPPH and ABTS radical-scavenging capacities, indicating that these two methods had similar predictive abilities with respect to antioxidant capacities [11]. It is interesting to investigate the structure-activity relationships for phenylethanoid glycosides **1–4**, which have similar structures, and the main differences are the substituents at C-3 (R1), C-4 (R2) and C-4′ (R3). It is inferred that the antioxidant activity of phenolics increases when there are free hydroxy groups in the molecule, which is consistent with the reported results [33]. Of the six flavonoids analysed (**5–10**), only luteolin (**5**) displayed strong antioxidant activities. In general, the antioxidant activities of flavonoids depend on the structure and substitution pattern of the hydroxy groups. The essential requirement for effective radical scavenging by flavonoids is a 3′,4′-*O*-dihydroxy B-ring structure, which confers higher stability in the radical form and participates in electron delocalization [34]; hence, luteolin (**5**) has a higher antioxidant capacity. The high radical scavenging capacity of esculetin (**12**) is probably due to the superior stability of radicals derived from catechol moieties compared to that of phenoxyl radicals [35]. The finding that acteoside (**1**) is a major component (0.803 g, 0.54%) of *C*. *cyrtophyllum* and the EAF subfraction demonstrated that compound **1** is a main active ingredient and is principally responsible for the significant antioxidant effect of *C*. *cyrtophyllum*.

**Table 2:** Antioxidant activities of phenolic compounds 1–12^a^.

Compound	1	2	3	4	5	6–11	12	VC
DPPH IC_50_ (μg/ml)	79.65±3.4	49.23±3.78	97.12±2.1	150.23±3.21	109.77±7.43		47.91±0.77	73.14±2.80
ABTS IC_50_ (μg/ml)	23.00±1.5	23.78±0.87	9.55±0.27	65.53±1.67	23.26±1.88		5.88±0.51	57.53±4.11

^*a*^ Responses are the means ± SD (n = 3).

## 4. Conclusions

Continuing our ongoing research into the antioxidant activity of the components of *C*. *cyrtophyllum*, we first demonstrated that its crude extracts and fractions of various polarity possess potential antioxidant and radical-scavenging activities through multiple mechanisms. EAF and BAF exerted the highest antioxidant effects. Fractionation of the EAF led to the isolation and identification of phenolic compounds **1–12**. Compounds **2–5** and **7–12** were obtained from *C*. *cyrtophyllum* for the first time. Compounds **1–5** and **12** exhibited considerable radical scavenging effects. Considering the yield, acteoside (**1**) is a main effective ingredient responsible for the DPPH and ABTS radical scavenging activities and could explain the significant antioxidant activities of the *C*. *cyrtophyllum* extracts, indicating it may be suitable as a natural antioxidant or alternative to toxic synthetic antioxidants in the food and pharmaceutical industries. Further work on the isolation and elucidation of other specific metabolites in *C*. *cyrtophyllum* responsible for the antioxidant activity is in progress in our laboratory, and *in vivo* biological tests should be conducted.

## 5. Ethics statement

This research did not include any human subjects or animal experiments.

## Supporting information

S1 File (DOCX)
