# Supplementary material for: Antioxidant activities of Clerodendrum cyrtophyllum Turcz leaf extracts and their major components
Source: PLoS One. 2020 Jun 23;15(6):e0234435. doi: 10.1371/journal.pone.0234435 (PMC7310832; doi:10.1371/journal.pone.0234435)
Supplement: S1 File — (DOCX) [file pone.0234435.s001.docx]

- 1. *Plant Material and Ethics Statement*

Fresh *C. cyrtophyllum* leaves were collected from the Extinct Crater Garden (E110°13′14″, N19°55′56″) on Hainan Island, China, in March 2013. The People’s Republic of China issued the specific permissions are required from authority of plant collection in a protected area of land, but not a national geological garden. Our plant materials were collected in a national geological garden and the author was not obliged to have any permissions. This work did not involve endangered or protected species, the species *C. cyrtophyllum* is a common plant growing nearby the curbside. A voucher specimen of the plant (P-DQ001) was deposited in the herbarium of the Institute of Tropical Agriculture and Forestry, Hainan University, China.

- 1. *Extraction Procedure*

Dried leaves of *C. cyrtophyllum* (150 g) were weighed and sieved (20 mesh) in an herb grinder (118 Swing, Zhejiang, China); the powdered samples were extracted twice according to a previous protocol [10]. The solvent was removed from the combined filtrates, and 61.44 g of ECE was obtained and redissolved in distilled water (500 ml). The solution was partitioned with 3×250 ml petroleum ether (60–90 °C), 3×250 ml dichloromethane, 3×250 ml ethyl acetate and 3×250 ml *n*-butanol. The resulting extracts were concentrated to yield 0.65, 5.53, 4.13, 13.28 and 36.97 g of the subfractions PEF, DMF, EAF, BAF and RF, respectively. The samples were stored at 4 °C. <http://dx.doi.org/10.17504/protocols.io.bdawi2fe> [PROTOCOL DOI]

- 1. *Antioxidant Activity*

The antioxidant activities of samples were determined using standard methods. VC and BHT were used as positive standards in the radical-scavenging assays. Gallic acid was used as a positive standard in the ferric reducing power assay. Ethylene diamine tetra acetic acid (EDTA) was used as a positive standard for the ferrous ion-chelating activity assay.

- - 1. *DPPH Radical-scavenging Activity.*

The DPPH radical scavenging activities were estimated [10] by mixing 0.1 ml of the extract with 3.9 ml of 60 μM solution of DPPH in ethanol. After 30 min of reaction, the absorbance was measured at 517 nm. The inhibition percent and 50% inhibition (IC_50_) values of DPPH radicals were calculated. http://dx.doi.org/10.17504/protocols.io.jiqckdw [PROTOCOL DOI]

- - 1. *ABTS Radical-scavenging Activity.*

The method described by [10] was used to determine the ABTS radical-scavenging capacity. An aliquot of extract (0.1 ml) was added to 3.9 ml of ABTS radical solution. The mixture was reacted for 30 min, and the absorbance at 734 nm was measured. The inhibition percent and IC_50_ values of the extracts for ABTS radical were calculated. http://dx.doi.org/10.17504/protocols.io.jirckd6 [PROTOCOL DOI]

- - 1. *Superoxide Radical-scavenging Activity.*

The superoxide radical scavenging effects were examined [11]. Briefly, 1 ml of the extract was added to 1 ml of 50 μM NBT solution, 1 ml of 468 μM NADH, and a 1 ml aliquot of 60 μM PMS reaction mixture. After 5 min, the absorbance was read at 560 nm. The inhibition percent and IC_50_ values were calculated. <http://dx.doi.org/10.17504/protocols.io.bdaxi2fn> [PROTOCOL DOI]

- - 1. *Hydroxyl Radical-scavenging Activity.*

The scavenging of hydroxyl radicals was determined following the method of Guo et al. [11] The reactions were performed with 0.3 ml of 20 mM sodium salicylate, 2.0 ml of 1.5 nM FeSO_4_, 1.0 ml of sample, and 1.0 ml of 6 mM H_2_O_2_. The reaction mixture was incubated for 1 h at 37 °C. The absorbance was measured at 510 nm. The inhibition percent and 50% of absorbance (EC_50_) were calculated. http://dx.doi.org/10.17504/protocols.io.bdazi2f6 [PROTOCOL DOI]

- - 1. *Reducing Power.*

The reducing power of the samples were assayed using the method of Guo et al.[11] Briefly, 1 ml of extract was added to 2.5 ml of phosphate buffer (0.2 M, pH 6.6) and 2.5 ml of 1% potassium ferricyanide. After 20 min, 2.5 ml of 10% trichloroacetic acid (TCA) was added, and then the mixture was centrifuged at 3000 rpm for 10 min. The upper layer (2.5 ml) was mixed with 2.5 ml of distilled water and 0.5 ml of 0.1% ferric chloride, and after 10 min, the absorbance was measured at 700 nm. The EC_50_ values were calculated from the graph of inhibition percentage against extract concentration. http://dx.doi.org/10.17504/protocols.io.bda2i2ge [PROTOCOL DOI]

- - 1. *Ferrous Ion-chelating Activity.*

The ferrous ion-chelating activities were determined according to Guo et al. [11] A 1 ml aliquot of extract was added to a solution of 100 µL of FeCl_3_ (2.0 mM), 3.7 ml of distilled water and 200 µL of ferrozine (5.0 mM). After 20 min, the absorbance was recorded at 562 nm. The inhibition percent and IC_50_ values were calculated. http://dx.doi.org/10.17504/protocols.io.bda4i2gw [PROTOCOL DOI]

- - 1. *Total Phenolic Content (TPC).*

The TPCs in the samples were determined by a colorimetric method based on the procedure described by Zhou et al. [10] Folin-Ciocalteu (FC) reagent (2 ml) was added to 2 ml of diluted extract. After 3 min, 750 µL of sodium carbonate anhydrous solution (7.5%, w/v) was added, and the mixture was adjusted to 10 ml with distilled water. After 2 h, the absorbance was recorded at 765 nm. Calibration curves were constructed with gallic acid as the standard at concentrations ranging from 0–100 μg/ml. http://dx.doi.org/10.17504/protocols.io.bda5i2g6 [PROTOCOL DOI]

- - 1. *Total Flavonoid Content (TFC).*

The amounts of total flavonoids were quantified [10]. The reaction mixture consisted of 1.0 ml of extract, 0.3 ml of 5% sodium nitrite and 4 ml of 60% ethanol. After 6 min, 0.3 ml of 10% aluminium nitrite was added. After 6 mins, 4 ml of 1 M sodium hydroxide solution was added. Then, the volume was brought to 10 ml, and the absorbance was measured at 510 nm. The TFC was calculated and is expressed as rutin equivalents (RE). A calibration curve was constructed with different concentrations of rutin (15–75 μg/ml) as a standard. http:// dx.doi.org/10.17504/protocols.io.bdbdi2i6 [PROTOCOL DOI]

*2.4. Isolation of the Antioxidant Metabolites from the EAF.*

EAF (4.13 g), which showed the strongest antioxidant activity, was subjected to silica gel column chromatography (CC), employing a step gradient of CH_2_Cl_2_-CH_3_OH (10:1, 10:2, 10:3, 10:5, 1:1, 0:1, v/v), and afforded eleven fractions (Fr. 1-Fr. 9) (Fig. 1). Fr. 2 was subjected to open silica gel CC using gradient elution with EtOAc-CH_3_OH (10:1-0:1, v/v) to yield fractions Fr. 2.1-2.4. Fr. 2.2 and 2.4 were separated using Sephadex LH-20 CC/ODS-HPLC to afford **6** (8.1 mg, 0.54 ‱) and **7** (20 mg, 1.33 ‱), respectively. Fr. 4 was subjected to polyamide CC using CH_2_Cl_2_-CH_3_OH-HCOOH as the eluent (10:2:1, v/v). Promising subfraction Fr. 4-3 was separated by RP C-18 CC eluted with CH_3_OH-H_2_O (1:1-1:0, v/v). Final purification was achieved by polyamide CC using CH_2_Cl_2_-CH_3_OH (10:4, v/v) to yield **1** (0.803 g, 0.54%). Fr. 5 was subjected to polyamide CC with CH_2_Cl_2_-EtOAc-CH_3_OH (5:5:1, v/v) as the eluent. Fr. 5-3 and Fr. 5-5 were separated using polyamide/RP C-18/Sephadex LH-20 CC to yield **5** (11 mg, 0.73 ‱), **11** (8 mg, 0.53 ‱) and **12** (5 mg, 0.33 ‱). Fr. 7.1, collected from Fr. 7 was subjected to polyamide CC with EtOAc-CH_3_OH (10:2, v/v), followed by ODS-HPLC using a gradient of CH_3_OH-H_2_O (3:7-9:1, v/v) as the eluent to yield **3** (10 mg, 0.66 ‱) and **9** (5 mg, 0.33 ‱). Fr. 8-1, Fr. 8-3 and Fr. 8-4, obtained from Fr. 8 with CH_2_Cl_2_-CH_3_OH (10:2, v/v), were separated using ODS-HPLC/Sephadex LH-20 CC to yield **2** (150 mg, 0.1%), **4** (5 mg, 0.33 ‱), **8** (3 mg, 0. 20 ‱) and **10** (8 mg, 0.53 ‱). http://dx.doi.org/10.17504/protocols.io.bda7i2hn [PROTOCOL DOI]
